# Diabetic nephropathy

**DOI:** 10.1186/1758-5996-1-10

**Published:** 2009-09-21

**Authors:** Themis Zelmanovitz, Fernando Gerchman, Amely PS Balthazar, Fúlvio CS Thomazelli, Jorge D Matos, Luís H Canani

**Affiliations:** 1 Endocrine Division, Hospital de Clínicas de Porto Alegre, Universidade Federal do Rio Grande do Sul, Porto Alegre, Brazil; 2 Universidade Federal do Rio Grande do Sul, Brazil; 3 Universidade do Sul de Santa Catarina, Brazil; 4 Medical School of Universidade Regional de Blumenau, Santa Catarina, Brazil; 5 Universidade Federal de Santa Catarina, Brazil

## Abstract

Diabetic nephropathy is the leading cause of chronic renal disease and a major cause of cardiovascular mortality. Diabetic nephropathy has been categorized into stages: microalbuminuria and macroalbuminuria. The cut-off values of micro- and macroalbuminuria are arbitrary and their values have been questioned. Subjects in the upper-normal range of albuminuria seem to be at high risk of progression to micro- or macroalbuminuria and they also had a higher blood pressure than normoalbuminuric subjects in the lower normoalbuminuria range. Diabetic nephropathy screening is made by measuring albumin in spot urine. If abnormal, it should be confirmed in two out three samples collected in a three to six-months interval. Additionally, it is recommended that glomerular filtration rate be routinely estimated for appropriate screening of nephropathy, because some patients present a decreased glomerular filtration rate when urine albumin values are in the normal range. The two main risk factors for diabetic nephropathy are hyperglycemia and arterial hypertension, but the genetic susceptibility in both type 1 and type 2 diabetes is of great importance. Other risk factors are smoking, dyslipidemia, proteinuria, glomerular hyperfiltration and dietary factors. Nephropathy is pathologically characterized in individuals with type 1 diabetes by thickening of glomerular and tubular basal membranes, with progressive mesangial expansion (diffuse or nodular) leading to progressive reduction of glomerular filtration surface. Concurrent interstitial morphological alterations and hyalinization of afferent and efferent glomerular arterioles also occur. Podocytes abnormalities also appear to be involved in the glomerulosclerosis process. In patients with type 2 diabetes, renal lesions are heterogeneous and more complex than in individuals with type 1 diabetes. Treatment of diabetic nephropathy is based on a multiple risk factor approach, and the goal is retarding the development or progression of the disease and to decrease the subject's increased risk of cardiovascular disease. Achieving the best metabolic control, treating hypertension (<130/80 mmHg) and dyslipidemia (LDL cholesterol <100 mg/dl), using drugs that block the renin-angiotensin-aldosterone system, are effective strategies for preventing the development of microalbuminuria, delaying the progression to more advanced stages of nephropathy and reducing cardiovascular mortality in patients with diabetes.

## Review

Diabetic nephropathy (DN) is the leading cause of chronic renal disease in patients starting renal replacement therapy [1] in the United States as well as in Brazil [2]. It is associated with increased cardiovascular mortality [2,3]. DN has been classically defined as increased protein excretion in urine. Early stage is characterized by a small increase in urinary albumin excretion (UAE), also called microalbuminuria or incipient DN [4-7]. More advanced disease is defined by the presence of macroalbuminuria or proteinuria. The latter is classically named overt DN. In most cases, proteinuria and decreased glomerular filtration rate (GFR) occur in parallel. Traditionally, GFR has been expected to decrease when proteinuria is established, but not before. However, it is clear today that some subjects could have DN without increased UAE [8,9]. About 10% of subjects with type 2 diabetes mellitus (DM) will have low GFR without micro- or macroalbuminuria [10]. This was also observed among patients with type 1 DM and microalbuminuria [11].

The prevalence of DN varies according to ethnicity: it is higher in African-Americans, Asians and Native-Americans than in Caucasians [1,12]. African-Brazilians are more susceptible to progress to end-stage renal disease (ESRD) than people of European ancestry, but there appears to be a similar prevalence of micro- or macroalbuminuria [13].

Among patients starting renal replacement therapy, the incidence of DN continued to rise from 1991 to 2001 [1]. This observation could not be attributed to older age or DM prevalence. From 1984-1996, the incidence of ESRD treatment attributable to DM (ESRD-DM) per 100,000 diabetic population increased in all age groups. However, in 1997-2002, ESRD-DM incidence decreased for people younger than 65 years (by 28% for those younger than 45 years and by 19% for those aged 45-64 years), did not change for those aged 65-74 years, and increased only among persons aged 75 years or older (by 10% from 350.3 to 383.7) . Although people younger than 65 years had the highest incidence of ESRD-DM prior to 1990, by 1999 their incidence was lower than in the older ones .

The increased incidence of ESRD attributable to DM suggests that other factors are involved in the etiology of DN, since a putative improvement in blood pressure (BP) levels, increased use of angiotensin converting enzyme (ACE) inhibitors and better glucose control due to lower glycemic targets have been frequent in recent years.

### Stages

According to UAE values, DN has been didactically categorized into stages. The cutoff values used [14] to characterize these stages are described in Table 1.

**Table 1:** Diabetic nephropathy stages based on urinary albumin excretion

**Stage**	**Urine with marked time** (μg/min)*	**24-hour urine** (mg/24 h)*	**Random urine sample**	
			**Albumin concentration** (mg/l)**	**Albumin/creatinine ratio** (mg/g)*

Normoalbuminuria	< 20	< 30	< 17	< 30
Microalbuminuria	20 -- 199	30 -- 299	17 a 173	30 -- 299
Macroalbuminuria	≥ 200	≥ 300	≥ 174	≥ 300

* Values according to the American Diabetes Association

** Gross et al., Diabetes Care 2005.

Although microalbuminuria is considered a risk factor for the development of macroalbuminuria, not all patients progress to this stage, and some may regress to normoalbuminuria [15,16]. The initial studies suggested that about 80% of type 1 diabetic patients with microalbuminuria would progress to proteinuria over a period of 6 to 14 years [4-6]. More recent studies suggest that only 30 to 45% of microalbuminuric patients will progress to proteinuria over 10 years of follow-up [15]. In fact, some of them will present regression to normoalbuminuria. This might be the result of more intensive glucose and BP control strategies employed in the last decade than in the initial studies. This regression of microalbuminuria is more frequent among subjects with short duration of microalbuminuria, glicohemoglobin A1c (HbA1c) below 8%, systolic BP <115 mm Hg, and favorable lipid profile (serum total cholesterol <198 mg/dl and triglycerides <145 mg/dl). Independent of the role as a prognostic factor for macroalbuminuria, the presence of microalbuminuria, reflecting a state of generalized endothelial dysfunction, is a risk factor for cardiovascular disease and mortality [17,18].

The cut-off values of urinary albumin to define the stages of DN are arbitrary (Table 1). On the one hand, not all subjects will progress to overt DN, and some might even regress as stated before. On the other hand subjects in the upper-normal range of albuminuria seem to be at high risk for complications. In patients with type 2 DM, the progression to micro- or macroalbuminuria is more frequent in individuals whose baseline UAE was normal but above 2.5 mg/24 h [19]. Furthermore, in another study after 10 years of follow-up, patients with type 2 DM and UAE values above 10 μg/min were at 29 times higher risk of developing DN [20]. Similar results were observed in patients with type 1 DM [21]. Another interesting observation is that patients with type 2 DM and UAE in the upper-normal range had higher BP than normoalbuminuric patients in the lower UAE range [22]. This favors the concept that UAE is a continuum similar to what has been demonstrated for BP and cholesterol levels.

In the microalbuminuric stage, no decline in GFR is expected. Once the subject has developed macroalbuminuria, the expected GFR decline is 1.2 ml/min/month in type 1 DM [23]. This could be decreased by BP treatment. In type 2 DM, the rate of GFR decline is less predictable. A mean decline of approximately 0.5 ml/min/month [24] has been described, but in some patients GFR may remain stable for long periods of time [25]. The greater GFR decline is associated with more advanced diabetic glomerulopathy and worse metabolic control [26].

### Screening and Diagnosis

The first step in screening for DN is to measure albumin in an isolated urine sample [27]. The results of albuminuria in an isolated sample can be expressed as albumin concentration (mg/l) or as albumin/creatinine ratio (mg/g) [28]. Although albumin concentration may be influenced by urine dilution/concentration, this measure appears to be the best choice, considering its cost and accuracy [29]. Every abnormal albuminuria test should be confirmed in two of three samples collected at a three to six-months interval, due to the daily variability of UAE [30]. Screening should not be performed under conditions that may increase UAE, such as hematuria, acute systemic diseases or fever, vigorous physical exercise, poor glycemic control, uncontrolled arterial hypertension and decompensated cardiac failure [31]. Bacteriuria had also been considered a factor that could interfere in the measure of urinary albumin [31-33]; but in a recent study this finding was not confirmed, suggesting that it is not necessary to exclude bacteriuria to measure albuminuria [34].

In situations in which UAE measurement is not available, semiquantitative dipstick measurements of albuminuria (for instance: Micral^® ^Test II) can be used, although these tests are less accurate [29].

The quantitative methods most commonly used to measure albuminuria are immunoturbidimetry, immunonephelometry and radioimmunoassay. However, recently it has been observed that an appreciable quantity of albumin is not detected by routine immunoassay methods, defined as non-immunoreactive fraction, which results in an underestimate of UAE [35-37]. On the other hand the HPLC (*high performance liquid chromatography*) method measures the immunoreactive and non-immunoreactive fractions which compose the total intact albumin, allowing the detection of earlier albumin elevations [37,38]. However, this method can overestimate UAE as observed in some community studies, possibly due to the fact that albumin peaks measured using this method can be confounded with other proteins [39]. Therefore, the significance of total intact albumin, both to diagnose DN, and for its association with cardiovascular disease has not yet been well established.

DN screening must be performed when DM is diagnosed in patients with type 2 DM, since these individuals may have had a silent form of DM for some time already. For patients with type 1 DM, it is recommended that screening be performed beginning in the fifth year after DM diagnosis or earlier if the DM is chronically poorly compensated, or if the patient is an adolescent. In all cases, if albuminuria is normal, screening must be repeated annually [27].

Although the measurement of albuminuria is essential to diagnose DN, there are some patients who present decreased GFR when UAE values are normal. Based on this, the classification of the *National Kidney Foundation *can also be used to stage chronic kidney disease in these patients (Table 2) [27]. It is recommended that GFR be routinely estimated for appropriate screening of DN. GFR can be measured using specific techniques such as inulin clearance, 51Cr-EDTA, 125I-iothalamate, and iohexol. However, in clinical practice, GFR is estimated by equations that take into account serum creatinine concentration and some or all of the following variables: age, sex, body weight and race. The equation recommended by the *National Kidney Foundation *is that of the study on Modification of Diet in Renal Disease (MDRD): GFR (ml/min/l.73 m^2^) = 186 × [serum creatinine (mg/dl) ^-1,154 ^× age (years) ^-0,203 ^× (0.742 if a woman) × (1.21 if African-American)]. If the creatinine measurement method is calibrated, the formula should use factor "175" in place of the value "186". The Cockroft-Gault formula, creatinine depuration (ml/min) = [140 - age (years)] × weight (kg)/[72 × serum creatinine (mg/dl)] × 0.85 (if a woman) is less precise [40]. The reference values of GFR for young individuals are 90 to 130 ml/min/l.73 m^2^, with the reduction of these values as age increases, in the order of 10 ml/min/decade after the age of 50 years [41].

**Table 2:** Chronic kidney disease stages

**Stage**	**Description**	**GFR (ml/min/1.73 m^**2**^)**
1	Renal damage* with GFR N or ↑	≥90
2	Renal damage* with GFR slightly ↓	60-89
3	GFR moderately ↓	30-59
4	GFR severely ↓	15-29
5	End stage chronic renal failure	< 15 or dialysis

*Renal damage is defined by abnormalities in the urine and blood tests, imaging exams or in pathology

GFR = glomerular filtration rate

Serum creatinine concentration should not be used as an isolated index for the evaluation of renal function, since its measure is affected by other factors besides GFR itself, such as tubular secretion, extrarenal generation and protein ingestion.

### Special Situations

Patients with micro- or macroalbuminuria, after the confirmation of diagnosis (2 measurements), should undergo a complete evaluation concerning differential diagnosis and assessment of renal function. DN is associated with several other conditions that need to be addressed making the management of these patients very complex. However this is not within the scope of the present manuscript and a detailed approach could be found in a recent review [30].

Diabetic patients can have other kidney diseases. The differential diagnosis is usually based on the history, physical examination, laboratory evaluation and kidney imaging. Renal biopsy has only been recommended in special situations. In the presence of micro or microalbuminuria and diabetic retinopathy in a patient with long term DM (e.g. >10 years) the assumption that DM is causing the renal disease is generally correct. Conversely, in diabetic patients with type 2 DM with a fast increment in albuminuria and in patients with type 1 DM where macroalbuminuria develop in the absence of diabetic retinopathy a differential diagnosis should be carried out. However, in type 2 diabetic patients the time of DM onset is usually unknown and retinopathy could be absent in a significant proportion (28%) of patients with albuminuria [42]. In summary, absent retinopathy, short duration of DM and faster decline in GFR and/or albuminuria increment are indications to suspect of nondiabetic renal disease [43]. If after a non-invasive evaluation the diagnosis is still unclear, a kidney biopsy should be discussed. In type 2 DM the prevalence of nondiabetic renal disease could vary from 12 to 38% [42,44,45]. All the kidney biopsy data are derived from retrospective studies. The differences in the prevalence of non-diabetic lesion observed in the studies probably reflect different criteria used to indicate renal biopsies. In one study, subjects with type 2 DM, gross proteinuria (>1 g) without retinopathy, and hematuria and no retinopathy, 19% of patients with confirmed DN had another glomerulopathy associated [45]. In this study, patients without diabetic glomerulosclerosis had a better prognosis than those with diabetic glomerulosclerosis [45]. Another aspect that needs to be addressed is that it is not clear if there is additional benefit of detecting other nephropathies in the management of these patients.

### Risk Factors

The two main risk factors for DN are hyperglycemia and arterial hypertension. However, DN develops in only about 40% of patients, even in the presence of hyperglycemia and elevated BP for long periods of time. This observation raised the concept that DN will develop only in a susceptible subset of patients [46-48]. Furthermore, family studies have confirmed a genetic contribution for the development of DN in both type 1 and type 2 DM [49-53]. Once DN is present, progression factors may act, favoring evolution to more advanced stages. There is evidence that some factors involved in the development of proteinuria are also common to the loss of GFR, but others are unique to each one of them [54].

#### Hyperglycemia

Hyperglycemia is a significant risk factor for the development of microalbuminuria, both in type 1 and in type 2 DM [21,55,56]. A reduction of 1% in HbA1c is associated with a 37% decrease in microvascular endpoints [57]. In the presence of micro- and macroalbuminuria the role of metabolic control is less defined, even though some studies showed a deleterious effect of high glucose levels on GFR [58,59]. Moreover, it was demonstrated that pancreas transplantation reversed renal damage in type 1 DM patients with mild to advanced DN lesions [60]. Recently a large trial also reinforced the importance of intensive treatment of DM to decrease the microvascular complications [61].

#### Arterial Hypertension

Arterial hypertension is a main risk factor for the development of DN [56,62], and probably the best known relevant factor related to its progression. Analysis of UKPDS showed that every 10 mmHg reduction in systolic BP is associated with a 13% reduction in the risk of microvascular complications, with the smallest risk among those patients with systolic BP <120 mm Hg [63].

#### Smoking

Smoking is a risk factor for DN [19,56] and might contribute to its progression [64]. Although some studies did not confirm these observations [55,59,65], it is strongly recommended to quit smoking in any phase of DN, also aiming to reduce the associated cardiovascular and cancer risk.

#### Dyslipidemia

In type 2 DM, elevated serum cholesterol is a risk factor for the development of DN [55,56]. In type 1 DM patients increased serum triglycerides, total and LDL-cholesterol were associated with micro- and macroalbuminuria [66,67]. High serum cholesterol also seems to be a risk factor for GFR loss in macroalbuminuric type 1 diabetic subjects [68].

#### Proteinuria

Proteinuria itself could lead to progression of DN. Proteinuria >2 g/24 h is associated with a greater risk of ESRD [69]. Increased leakage of albumin may induce glomerular damage probably through activation of inflammatory cascades [70]. This would be a reason to target decreased urinary albumin excretion in DN treatment.

#### Glomerular hyperfiltration

Elevated GFR values are present in about one third of type 2 DM patients [71,72] and theoretically it could cause DN due to glomerular damage [73]. Studies led to controversial findings regarding its role as a risk factor for the development of DN [20,71,74]. Type 2 DM patients with a single-kidney more often present increased UAE levels [75,76]. On the other hand, type 1 DM patients with only one kidney do not have a more aggressive disease [77]. Glomerular hyperfiltration probably plays a small role, if any, in the development of DN [78].

#### Dietary factors

Increased dietary protein intake seems to be associated with the presence of higher UAE values, at least in patients with type 1 DM [79]. In patients with type 2 DM this association has not been documented. The source of proteins in the diet also seems to be related to the presence of DN. A higher intake of fish protein is related to a lower risk of microalbuminuria in type 1 DM patients [80]. The mechanisms involved in these findings are unknown but probably related to hemodynamic factors [81].

Regarding the dietary lipid content, an association has been observed between the higher intake of saturated fat and the presence of microalbuminuria in patients with type 1 DM [82]. In patients with type 2 DM, very recently, it was observed that the presence of microalbuminuria was associated with the lower content of polyunsaturated fatty acids, especially those of vegetal origin [83]. In a study performed with patients with type 1 and type 2 DM, followed for 6 years, it was also demonstrated that those who evolved with regression of the DN presented a higher intake of polyunsaturated fatty acids and a lower intake of saturated fatty acids [84].

#### Genetic risk factors

The exact genetic model underlying DN susceptibility is uncertain, but theoretically few genes with a major contribution and some with minor interaction with the environment could cause DN [47,48]. Unfortunately, no gene with a major effect had been identified so far. The knowledge of which gene(s) predisposes to DN will allow the identification of patients at high risk for this complication, and adoption of preventive measures.

In genetic studies the clear definition of the phenotype, DN, is very important. DN could be defined by different parameters: for instance, the presence of microalbuminuria, macroalbuminuria, ESRD or decreased GFR. Some genes probably are involved in the development of proteinuria, others with decline in GFR and some will be involved in both situations [47,48]. Therefore, a more comprehensive definition of DN used in the genetic studies is important to make the results more comparable.

A familial aggregation of DN has been demonstrated in studies of sibling-pairs [49,53,85], parent-offspring pairs or studies of extended families [51,52]. One practical application of the studies with diabetic siblings is that the chance of having DN increases 2-3 times if the subject's sibling has DN when compared to the subject who has a normoalbuminuric sibling, either in type 1 or type 2 diabetes [49,53].

Recent advances in technology make easier to look for regions in whole genome linked to different DN phenotypes [86-88]. This approach identified regions and putative genes not previously known to be associated with DN and it could raise new candidate genes. Moreover, new targets for drug development may come into sight, since some of the genes found are novel and have not been previously implicated in the pathogenesis of DN.

Association studies of candidate genes have been performed aiming to identify polymorphic variants associated with DN or with different degrees of renal disease. Often, genes that play a role in the expression of proteins that are related to the modulation of cytokines, proteins involved in the glycid and lipid metabolism, in the formation of extracellular matrix, in blood pressure homeostasis, and in insulin sensitivity, have been considered candidates for the development of DN [9,30,89]. However, the studies have not been successful in identifying genes that consistently show an association with DN. Replication studies have demonstrated conflicting results [90]. The evaluation of 360 thousand polymorphisms in patients with type 1 DM, with and without DN, showed a total of 13 polymorphisms located at 4 loci in two independent cohorts of subjects strongly associated with the presence of DN [87]. Some of these polymorphisms are located in genes highly expressed in the kidney with DN, and its development over time [87].

Another approach that has been used to investigate the genetics of DN involves the study of microRNAs role on this process. These are non-encoding short RNAs that induce post-transcriptional protein modifications. Little is known about these molecules and their role in DN. In a study, microRNA mirR-192 expression was increased in the glomeruli of rodents with DM [91]. Their induction by TGF-β in mesangial cells caused increased collagen synthesis and suggests that this type of molecule may be implicated in the development of DN, opening up a new prospect of research in elucidating the pathogenesis of this DM complication. The replication of this finding and this type of approach must be better explored in studied conducted in human beings.

As previously stated, Brazilians of African descent have more aggressive renal disease than people of European ancestry [13]. This could be due to several reasons, such as the presence of different risk factors, different access to medical attention, and socioeconomic differences. However, none of the assessed known risk factors were different between African and Europeans [13,92] make unclear an explanation for the different rates of DN between black and white subjects. Unfortunately, data on socioeconomic status were unavailable. An alternative explanation for this observation, but hard to prove, would be a different genetic susceptibility.

### Pathology

DN in individuals with type 1 DM is initially characterized by a thickening of the glomerular and tubular basal membrane, with progressive mesangial expansion leading to the progressive reduction of the glomerular filtration surface [93]. Concurrent interstitial morphological alterations also occur, as well as hyalinization of the afferent and efferent glomerular arterioles [93]. Mesangial expansion can be diffuse (diabetic glomerulosclerosis) or with areas of marked mesangial expansion, forming roundish and fibrillar zones, with nuclei in palisade (nodular glomerulosclerosis, Kimmelstiel-Wilson nodes). While mesangial expansion is the critical lesion which leads to progression to loss of renal function, damage to the tubular glomerular junction, to the tubules and to the interstice determines progression to ESRD [87,91].

Podocytes damage also appears to be involved in the glomerulosclerosis process. In a study conducted in Pima Indians, highly susceptible to developing DN, a smaller number of podocytes per glomerulus was the greatest predictor of increased UAE and progression to clinical DN [93]. When this finding was present, normoalbuminuric individuals had a higher risk of progressing to renal disease than those who did not have a podocyte lesion [93]. In addition, nephrine, a protein synthesized by the podocyte and considered vital to the stability of the glomerular barrier, has its expression reduced in DN [94]. The administration of ACE inhibitors results in the expression of nephrine at levels similar to those of individuals with DM without DN [95].

In a subgroup of patients with DM, loss of renal function precedes the development of microalbuminuria. This group presents more advanced glomerular lesions than those that present microalbuminuria [93].

Renal lesions in individuals with type 2 DM are more complex than in individuals with type 1 DM. The prevalence of a renal lesion that is non-typical for DM in individuals with type 2 DM is high, reaching 10 - 30% of subjects with proteinuria [91,93]. In a minority, the histopathological aspects are similar to the typical lesion of subjects with type 1 DM. The rest presents only mild or absent DN, with or without tubulointerstitial alterations, arteriolar alterations or diffuse glomerulosclerosis [93]. The tubulopathy is possibly related to persistent hyperglycemia and changes related to age, atherosclerosis and arterial hypertension [91]. Despite the heterogeneity of the lesions and the impact of diseases such as arterial hypertension on individuals with type 2 DM, in a large cohort of individuals with type 2 DM, the severity of the lesions was correlated with the progression of DN and the velocity of GFR loss [26].

### Pathophysiological Mechanisms

#### Hemodynamic factors

In an initial phase, DN is characterized by glomerular hyperfiltration due to a reduction in the resistance of the afferent and efferent glomerular arterioles, and consequent increased renal perfusion. Although the mechanisms that lead to glomerular hyperfiltration are unclear, obesity and the release of a number of proinflammatory factors and growth factors that occur in DM appear to have a role [96,97]. In a study performed by our group, the levels of endothelin 1 (ET-1), an important vasoconstrictor, were correlated with UAE, and its plasma levels were progressively higher according to the higher degree of DN [98]. This early defect in autoregulation of renal perfusion makes it easier for albumin to leak from capillaries to renal glomerulus, and leads to compensatory increase of mesangial matrix, thickening of the glomerular basement membrane and podocyte damage. Albuminuria also activates a series of inflammatory pathways through tubular cells and feeds this process [91]. In addition, the mechanical stress resulting from renal hyperperfusion induces the release of cytokines (TNF-α), growth factors (VEGF, TGF-β1), cholesterol and local triglycerides that induce the accumulation of proteins from extracellular matrix, leading to mesangial expansion and glomerulosclerosis. A reduction of TGF-β1 by blocking the renin-angiotensin-aldosterone system retards the progression of DN and preserves glomerular morphology [99].

#### Hyperglycemia and advanced products of non-enzymatic glycosilation

Persistent hyperglycemia is a strong risk factor for DN and causes the proliferation of mesangial cells and their matrix, as well as the thickening of the basement membrane. Hyperglycemia increases the expression of *vascular endothelial growth factor *(VEGF) in podocytes causing increased vascular permeability. Hyperglycemia also increases the generation of advanced products of non-enzymatic glycosilation of proteins through activation of aldol reductase pathway and protein kinase C (PKC). The final products of non-enzymatic glycosilation are bound to collagen and proteins that constitute the glomerular basement membrane and make the glomerular barrier more permeable to the passage of proteins, resulting in increased UAE [94,100-104].

#### Cytokines

A series of circulating markers of inflammation such as C reactive protein and interleukin 1, 6 and 18, and tumor necrosis factor are increased in DN and their levels correlate with albuminuria and progression to ESRD. In addition, hyperglycemia, TGF-β1 and angiotensin II stimulate the secretion of VEGF, causing the production of endothelial nitric oxide, vasodilation and glomerular hyperfiltration [94]. Hyperglycemia, possibly mediated by oxidative stress, also induces angiotensin II to the synthesis of TGF-β, type IV collagen and fibronectin, contributing to progressive glomeruloesclerosis [87].

Inflammatory factors are also involved in the development of tubulointerstitial lesion, and appear to lead to accumulation of macrophages in the tubular interstice in animal models designed to study DN. Macrophages also produce free radicals, inflammatory cytokines and proteases that induce tubular damage [91]. Furthermore, glomerular and renal cells also produce a series of inflammatory factors when they are exposed to glomerular hyperfiltration and increased UAE, intensifying this process [91].

### Treatment

The principles of prevention and treatment of DN are the same. However, the role of each factor could be different in each stage of disease. It is important to define the DN stage that is the target of intervention (microalbuminuria, proteinuria or GFR) and the outcome of interest. Two recent meta-analyses have demonstrated different results when evaluating different outcomes, such as proteinuria, GFR decline or progression to ESRD [105,106]. Both, ACE inhibitors and angiotensin receptor blockers (ARBs) seem to be effective reducing proteinuria and decreasing the creatinine doubling rate, but not decreasing mortality [106]. Probably the best treatment is a multiple risk factor interventional approach, but due to a practical point of view each aspect will be addressed individually. The goal to be pursued is retarding the development or progression of DN and to decrease the subject's cardiovascular risk and mortality.

In normo- or microalbuminuric subjects, the aim of treatment is to intervene at arterial hypertension, hyperglycemia, smoking habit and probably dyslipidemia. Even in the absence of clear data showing that the management of these risk factors individually is beneficial to DN, they are also risk factors for cardiovascular disease and should be aggressively treated [30].

Clinical trials have demonstrated that intensive treatment of hyperglycemia is associated with a decreased risk for the development of DN in type 1 and type 2 diabetic patients [107-110]. In type 1 and type 2 subjects the effect of intensive therapy could be seen many years later [108,111]. The effect of the intervention in hyperglycemia in type 1 macroalbuminuric subjects is not so clear [107,112,113]. This became more evident in the EDIC/DCCT follow up study [108]. In the Kumamoto study, prevention of macroalbuminuria was observed in type 2 DM patients intensively treated [110].

Recent studies designed to evaluate the benefit of intensive glycemic control in large sets of patients showed a minor protective effect on the development of progression of albuminuria [61,114]. In the Intensive Blood Glucose Control and Vascular Outcomes in Patients with Type 2 Diabetes (ADVANCE) trial, the group in the intensive arm for an average of 5 years showed a small reduction in the number of cases with new-onset microalbuminuria compared to the standard therapy group (23.7 vs. 25.7%) [61]. No effect was observed in the serum creatinine values [61]. The same was observed in the Glucose Control and Vascular Complications in Veterans with Type 2 Diabetes (VADT) study [114]. Patients in the intensive arm for a mean of 5.6 years did not show any benefit regarding changing serum creatinine or GFR values [114] and a minor effect on albuminuria levels was observed [114].

Treatment of hypertension leads to an important risk reduction in cardiovascular and microvascular events. In the UKPDS, a reduction from 154 to 144 mm Hg on systolic BP reduced the risk for the development of microalbuminuria by 29% [115]. BP targets for patients with DM are lower (130/80 mm Hg) than those for patients without DM [116]. In the Hypertension Optimal Treatment (HOT) study a reduction of diastolic BP from 85 to 81 mm Hg resulted in 50% reduction in the risk of cardiovascular events in diabetic but not in non-diabetic patients [117]. In the presence of microalbuminuria the treatment of hypertension, irrespective of the agent used, produced a beneficial effect on albuminuria [118].

Aggressive treatment of hypertension should be established in subjects with DM. A discussion of agents used to treat hypertension in patients with DN are beyond the scope of this manuscript, and recent guidelines [108,116] and excellent reviews in this subject are available [30,119,120].

In order to reach the BP goal of 130/80 mmHg in diabetic patients in general [116] or 125/75 mmHg in patients with proteinuria >1.0 g/24 h and increased serum creatinine, three to four antihypertensive agents are usually necessary [121].

The choice of anti-hypertensive agents to use is in some way not a problem in clinical practice, because to reach the BP goals the majority of patients will need several agents. However, due to the known renoprotective effect of ACE inhibitors and ARB, these agents (see below) should be used initially associated with a diuretic.

#### Renin-Angiotensin System (RAS) Blockade

ACE inhibitors could be used in normotensive subjects to prevent or postpone the development of microalbuminuria [122]. The aim of ACE inhibitors and ARBs use is not only to diminish the risk for the development of micro- and macroalbuminuria [123-125] but also to decrease the occurrence of cardiovascular events [124]. However, a recent 5 year multicenter randomized controlled trial involving 285 normoalbuminuric, normotensive patients with type 1 DM failed to show any improvement in biopsy parameters with losartan (100 mg daily) or enalapril (20 mg daily) compared to placebo [126]. Surprisingly, the 5-year cumulative incidence of microalbuminuria was 17% with losartan, significantly greater than with placebo (6%, P = 0.01). The enalapril group had a similar incidence of microalbuminuria (4%, P = 0.96) in comparison to the placebo group [126].

RAS blockade with ACE inhibitors or ARB confers an additional benefit on renal function. This renoprotective effect is independent of BP reduction [118,127]. These drugs decrease UAE and the rate of progression from microalbuminuria to more advanced stages of DN. A meta-analysis of 12 trials in non-hypertensive microalbuminuric type 1 diabetic patients showed that ACE inhibitors decreased the risk of progression to macroalbuminuria by 60%, and increased the chances of regression to normoalbuminuria [128]. Therefore, the use of ACE inhibitors or ARB is recommended for all microalbuminuric patients, even if normotensive [14]. ARBs were also effective in reducing the development of macroalbuminuria in microalbuminuric type 2 diabetic patients [127,129].

The aggressive treatment of hypertension has a strong beneficial effect in reducing GFR decline in proteinuric type 1 diabetic patients [130]. This reduction in GFR decline was predicted by reduction in albuminuria [131]. According to the MDRD trial, the lower the BP the greater the preservation of renal function in non-diabetic patients [132]. Patients with proteinuria >1 g/day and renal insufficiency had a slower decline in renal function when BP was <125/75 mm Hg [132]. Addition of ACE inhibitors in proteinuric type 1 [133] or ARB in macroalbuminuric type 2 [134,135] diabetic patients has a beneficial effect in decreasing proteinuria and reducing renal function decline. The effect of ARBs on protein excretion could be noted within 7 days after starting the treatment, and may persist after [136]. It seems to be independent of BP reduction [127] and has a dose response effect beyond the doses needed to control BP [137]. An acute increase in serum creatinine up to 30 to 35% that stabilizes within 2 months might occur and it is not a reason to stop the treatment [138]. Increase in serum creatinine above these values should raise the possibility of renal-artery stenosis [138,139]. Another limitation to the use of ACE inhibitors is hyperkalemia, especially among those with renal insufficiency [138]. Acute hyperkalemia (>5.5 mEqL) is an indication to stop these medications. Therefore, albuminuria, serum creatinine and potassium should be checked monthly in the first 2 to 3 months after starting treatment with ACE inhibitors or ARB [138,139].

ACE inhibitors and ARB interrupt the RAS at different levels, and the combination of these classes of drugs (RAS dual blockade) has been proposed [140] as an alternative to treat DN. It has been suggested that this association would have an additive effect on renoprotection. The combination of ARB and ACE inhibitors are effective in reducing UAE ratio in hypertensive patients with type 2 DM when compared to each drug alone. However, this is also associated with lower BP values in the group that used both drugs [140,141]. A recent large trial (ONTARGET) in diabetic and nondiabetic subjects showed that the association of the two classes of drugs had a major effect on decreasing proteinuria but not on GFR decline or mortality [142]. In fact, a worse effect on GFR and mortality was observed. Analyzing the subgroups, the increased mortality came from the less sick subjects. Among diabetic subjects no increased mortality was observed, but also no benefit from the dual blockage was observed [142]. The VA NEPRHON-D study aimed to evaluate this issue is patients with type 2 DM [143].

Another step that has been proposed to be blocked is the aldosterone action. Adding the aldosterone antagonist - spironolactone - to ARBs [144] or ACE inhibitor [145] is also more effective in reducing UAE and BP in type 2 diabetic patients than each drug alone. A recent meta-analyses that included diabetic and non-diabetic subjects demonstrated that the addition of aldosterone antagonists in patients already on ACE inhibitors and ARBs reduces proteinuria in chronic kidney disease [146]. This was not associated with an improvement on GFR, but increases the risk of hyperkalemia. Long-term effects of these agents on renal outcomes, mortality, and safety need to be determined [146].

More recently, the dual blockage of the renin-angiotensin-aldosterone system with aliskiren, a direct renin inhibitor, and losartan at maximal recommended dose (100 mg daily) showed a greater reduction in proteinuria (20%) compared to losartan and placebo [147]. The effect does not seem to be due to anti-hypertensive effect. However, this was a short duration study (12 weeks) and long term studies are needed. The ongoing trial ALTITUDE might answer some of these questions [148]. This placebo controlled, randomized trial intends to follow-up about 8600 subjects during two years and compare the effect of aliskiren added to standard treatment (ACE or ARBs) [148]. The results will be available by 2012.

#### Hyperglycemia treatment peculiarities

The treatment of DM is not the aim of the present review, but a few special remarks could be made regarding the treatment of hyperglycemia in a patient with renal disease (Table 3).

**Table 3:** Treatment of hyperglycemia in the patient with type 2 diabetes mellitus and chronic kidney disease

				**Stage of Renal Disease**		
				
	**Clearance**	**Reduction of HbA1c**	**Risk of hypoglycemia**	**III**	**IV**	**V**
Glibenclamide [153,154]	Hepatic metabolism: 100%. Excretion: bile and feces 50% and urine 50%	-1.5%	High (active metabolites)	Avoid	Avoid	Avoid
Glipizide [154]	Excretion: metabolites 90% in urine and feces. 10% excreted without metabolization	-1.5%	Low	Can be used	Can be used	Can be used (adjustments)
Glimepyride	Hepatic metabolism 100%. Excretion: urine 60% and feces 40%	-1.5%	Low	Can be used	Can be used	Use with care
Repaglinide [155,157]	Hepatic metabolism: 100%. Excretion: 10% urine and 90% feces	-1.0%	Low	Can be used	Can be used	Use with care. Adjust dose
Nateglinide [156,158,159]	Hepatic metabolism: 85%. Excretion: urine 83% and feces 10%. 15% excreted inactive in urine	-0.7%	High (active metabolites)	Use with care	Use with care	Avoid if possible
Acarbose* [153,160]	Excretion: urine 34%, feces 51% and <2% in urine in the free or active metabolic form	-0.6%	Low	Can be used	Can be used	Avoid
Rosiglitazone [162]	Hepatic metabolism and excretion in the urine, of rather inactive metabolites in the urine 64% and feces 23%	-0.6 to 1.5%	Low	Can be used	Can be used	Can be used
Pioglitazone [162]	Hepatic metabolism and excretion in urine of rather inactive metabolites in the urine 15% and feces 85%	-0.6 to 1.5%	Low	Can be used	Can be used	Can be used
Sitaglipitine [171,172]	Excretion: urine 87% and feces 13%, in an unaltered form.	-0.7%	Low	Can be used	Can be used. Reduce dose 50%	Can be used. Reduce dose 75%
Vildagliptine	Excretion: urine: 85% and feces 15%.	-0.7%	Low	Can be used	Can be used	Not recommended
Exanetide [173]	Metabolism and renal excretion	-1.0%**	Low	Can be used	Not recommended	Not recommended

Metformin is the standard therapy for patients with type 2 DM and will only be briefly discussed here. Metformin is contraindicated when serum creatinine is above 1.5 mg/dl in men and 1.4 mg/dl in women due to the increased risk of lactic acidosis [149]. However, these values are being questioned [150]. In these creatinine ranges, some subjects will be using metformin on chronic renal disease stages II and III [151].

Sulfonylureas and their metabolites, except glimepiride, are eliminated via renal excretion and should be used with caution in patients with GFR [152]. Glibenclamide is a potent drug and has been known for a long time. It is low cost and available in the public health system. However, it presents a high risk of hypoglycemia. It has active metabolites that increase in patients with decreased GFR, and its pharmacological action is such that the use of glibenclamide is not recommended from stage 3 onwards [153,154]. Among the sulfonylureas there is also glipizide which carries a lower risk of hypoglycemia being and alternative on this situation [154]. Glipizide can be used in chronic renal disease stages 3 and 4. It could still be used in stage 5, with a therapeutic adjustment. Glimepiride is a third generation sulfonylurea with a slightly higher cost and a lower risk of hypoglycemia. However, it is believed that it has a few active metabolites filtered by the kidneys what could be potentially related to higher risk of hypoglycemia compared to glipizide.

Repaglinide [155] and nateglinide [156] have a short duration of action, are excreted independently of renal function and have a safety profile in patients with renal impairment. These drugs, like the sulfonylureas, are insulin secretagogues, but they act in different cellular membrane channels, and this brings some pharmacological properties such as quick initial action, non-prolonged action and greater effect on post-prandial glycemia. A flexible aspect of this drugs making therapeutic management easier is the lower risk of hypoglycemia because of the different connection of the membrane channels. But one side effect described similar to observed with the sulfonylureas is weight gain. Its cost is higher than that of sulfonylureas, but theoretically it has a less deleterious effect on beta cells. Among the glinides, the first choice would be repaglinide because of the low risk of hypoglycemia, and it can be used in stage 3 and stage 4 [155,157]. Data in the literature are not sufficient to indicate the use of this drug in chronic renal disease stage 5. Nateglinide would be at a disadvantage because it is less potent, and it has active metabolites that can increase the risk of hypoglycemia in subjects with decreased GFR [158,159].

Acarbose is a drug that is not potent to reduce HbA1c. However, as its pharmacological action principle is the inhibition of enzyme alpha glycosidase in the small bowel, reducing glucose absorption in the gastrointestinal tract, it is a useful drug to adjust post-prandial hyperglycemia. The metabolism of this drug is practically 100% gastrointestinal, part is excreted in the urine and most of it in the feces, and a small form is excreted in the form of the active metabolite [153,160]. The concern of using acarbose in subjects with chronic kidney disease is the accumulation of these metabolites that may lead to hepatic lesions. Thus, acarbose would be contraindicated in subjects with chronic renal disease. There are insufficient data in the literature to use this drug with a creatinine greater than 2 mg/dl. It could be considered up to stage 3, and it should be avoided in stages 4 and 5 [153,160].

Glitazones, represented by rosiglitazone and pioglitazone, act through the PPR gamma system and are insulin sensitizer drugs that increase the muscle uptake of glucose and diminish the atherogenic profile of the DM patient, and could be used in renal failure [161,162]. Rosiglitazone has been shown to decrease UAE in type 2 diabetic patients as compared to glyburide, suggesting a beneficial effect in the prevention of renal complications of type 2 DM [163]. This antiproteinuric effect occurs also in nondiabetic disease [161,164,165]. The side effects include anemia, water retention, weight gain and potential hepatotoxicity due to the accumulation of its metabolites. Recently, cardiovascular safety and the risk of increased incidence of fractures have been discussed [166,167]. Both would present a low risk of hypoglycemia and could theoretically be used in the different stages of chronic renal disease without adjusting the dose [168].

A recent meta-analysis suggests beneficial effects of glitazones, with improvement of dyslipidemia in DM, internal carotid intima layer thickness reduction, improved fibrinolysis, and a direct action of the PPR gamma system at glomerular, tubular and vascular levels [169]. In theory, all these actions (hemodynamic, anti-inflammatory, anti-proliferative and metabolic) would be beneficial actions in nephropathy [169].

In relation to fractures, a recent meta-analysis showed that in the female population there has been up to two-fold increase in the incidence of fractures including both hip and vertebral fractures [170]. Since a uremic patient has already an increased osteometabolic risk, a drug that would increase the incidence of fractures should be questioned in these patients.

Two representatives of the DPP-4 inhibitors are available, vildagliptin and sitagliptin. These drugs inhibit the dipeptidyl peptidase-4 enzyme which, in turn, prevents degradation of the GLP-1 which remains active longer. Thus they lead to the reduction of fasting and post-prandial glycemia, without a risk of hypoglycemia. The gliptins suppress the high release of glucagon and are neutral as regards weight. The side effects include airway infection and transaminases elevation. The standard dose is 100 mg, orally, in a single daily dose. Sitagliptin secretion occurs mostly in urine and an adjustment in the dose is recommended according to the stage of renal disease: 50 mg for stage 3 and 25 mg for stages 4 and 5 [171,172]. Vildagliptin also is predominantly excreted in the urine. It is unnecessary to adjust the dose in patients with mild or moderate renal failure (50 mg orally, bid). The use of vildagliptin is not recommended, according to the directions that accompany medications, in patients with severe renal failure, patients who are already on dialysis or some other renal substitution therapy.

Exenatide is a GLP-1 analog. Subcutaneous applications (beginning at 5 μg bid for 30 days and then 10 μg bid) should be performed up to one hour before meals twice a day. It is a drug that reduces weight, which may be an advantage in managing the diabetic patient. The major side effects are nausea and vomiting, what occasionally an individual cannot tolerate using it. It is metabolized and excreted by the kidneys. It presents a low risk of hypoglycemia and can be used in stage 3, and it is not recommended in stages 4 and 5 due to the increased risk of side effects [173].

However, when the renal function is highly compromised, metformin, exenatide and gliptins are contraindicated, and insulin secretagogues are usually not very effective, since these patients have low endogenous production of insulin. Therefore, most patients should be treated with insulin [153]. We should remember that the half life of insulin is changed as soon as the individual begins to have a major renal function impairment. Pharmacokinetics is modified, and the insulin will have a slightly longer profile. This may make it difficult to manage the day to day situation, in peculiar situations on different days, i.e., the individual who is undergoing a dialysis session may feel bad and change his diet on that day. We should be able to rationalize more with the flexibility of doses here, which is often rather difficult for the patient and the physician.

These individuals certainly will have a greater propensity to hypoglycemia, so we have to be more careful and remember that hypoglycemia may be one of the complications implicated in the increased cardiovascular mortality of these patients. We should also keep in mind that therapeutic goals should be individualized.

#### Dietary intervention

There are several modalities for a dietary intervention in DN, whether changing protein content or through the manipulation of lipid content. However, few have their efficacy shown based on long term randomized clinical trials.

In patients with type 1 DM, in different stages of renal disease, protein restriction in the diet has shown that it can reduce the decline of renal function and albuminuria. According to a meta-analysis of studies performed with type 1 DM patients and clinical nephropathy, dietary protein restriction retards DN progression [174]. However, several of these studies were randomized with a crossover design, and the maximum time of follow up was 36 months. Besides, in these studies there was no evidence of benefit on hard outcomes such as mortality or risk of end stage chronic renal failure. A randomized controlled clinical trial with patients with type 1 DM and DN followed for four years, showed that a diet with a moderate protein restriction (0.9 g/kg/day) was associated with a 76% reduction of the risk of end stage chronic renal failure or death [175].

In patients with type 2 DM this benefit has not been well established. There are few studies with type 2 DM patients addressing this issue, showing no benefit on renal function, probably due to lack of compliance with the diet and short follow-up [176]. A recent meta-analysis performed with eight studies including patients with type 1 and 2 DM showed a benefit of protein restriction on proteinuria reduction, but not on GFR reduction [177]. The American Diabetes Association recommends moderate protein restriction (0.8-1.0 g/kg/day) for patients in the initial stages of DN, and a reduction to 0.8 g/kg/day for patients in a more advanced stage of this complication [27].

Interventions in the dietary lipid content has also been suggested, especially by manipulating the type of meat in the diet. Substituting red meat by chicken meat in the diet over the short term proved be able to reduce UAE, and also the serum levels of total cholesterol, LDL and apolipoprotein B in patients with type 2 DM and micro and macroalbuminuria [89,178]. Recently it was also observed that the beneficial effect of this dietary intervention on renal function was similar to the use of enalapril for a 12-month period in patients with type 2 DM [179]. This effect is probably related to the lower saturated fat content and greater proportion of polyunsaturated fatty acids, observed in chicken meat compared to red meat. Long term studies are needed to confirm this favorable effect.

#### Dyslipidemia

The desired target of LDL is <100 mg/dl for patients with DM in general, and <70 mg/dl when cardiovascular disease is present. No data based on a large clinical trial is available showing that the treatment of dyslipidemia is able to prevent the development or progression of DN. In the *Heart Protection Study *(HPS), sinvastatin, 40 mg, reduced vascular event rates and GFR decline in patients with DM by 25%, independent of baseline cholesterol levels. Furthermore the results of the Collaborative Atorvastatin Diabetes Study (CARDS) demonstrated a marked reduction in cardiovascular events in DM patients, and at least one additional risk factor for coronary disease, suggesting that all DM patients should use statins. A recent publication of CARDS showed a modest beneficial effect of atorvastatin on eGFR, particularly in those with albuminuria [180]. However, atorvastatin did not influence albuminuria incidence [180].

#### Multifactorial intervention

As stated before, probably the best approach to a subject with DN is a multifactorial intervention. However, only one study, with a small number of patients (n = 160) addressed this aspect [181]. In this study the targets were: BP levels <130/80 mm Hg, fasting serum cholesterol <175 mg/dl, fasting serum triglycerides <150 mg/dl, and HbA1_c _<6.5%. The intervention consisted of a stepwise implementation of lifestyle changes and pharmacological therapy including low-fat diet, three to five times a week light-to-moderate exercise program, smoking-cessation course, and prescription of ACE inhibitors or ARB and aspirin. The multiple intervention group had a 61% reduction in the risk of macroalbuminuria, and a 58% and 63% reduction in the risk of retinopathy and autonomic neuropathy, respectively. Most importantly, a 55% reduction in the risk for the development of a composite end-point consisting of death from cardiovascular causes, non-fatal myocardial infarction, revascularization procedures, non-fatal stroke and amputation was also associated with the multifactorial intervention. It is important to point out that even among highly motivated subjects only a small number reached the proposed goals. Less than 20% in the intensive arm reached the HbA1c goal and less than 50% the systolic BP goal [181].

## Conclusion

Diabetic nephropathy is a chronic complication of DM with a growing incidence. Therefore it is essential to have a better understanding of it, especially in relation to prevention and aggressive management to avoid progression to ESRD. Besides, its direct association with cardiovascular complications makes it imperative to perform intensive, early management of the risk factors. The study of DN has evolved a lot as regards its pathophysiology, stages of renal involvement and, especially, the therapeutic instruments available. Early detection of DN, the multifactorial approach targeting the main risk factors (hyperglycemia, hypertension, dyslipidemia and smoking), and the use of renoprotective agents such as the drugs that act on the renin-angiotensin-aldosterone system, may delay progression of kidney disease in DM, besides reducing cardiovascular mortality.

## Abbreviations

**ACE**: angiotensin convertin enzyme; **ARB**: angiotensin receptor blocker; **BP**: blood pressure; **DM**: diabetes mellitus; **DN**: diabetic neprhopathy; **ESRD**: end-stage renal disease; **ET-1**: endothelin-1; **GFR**: glomerular filtration rate; **HbA1c**: glicohemoglobin A1c; **MDRD**: Modification of Diet in Renal Disease; **RAS**: renin-angiotensin system; **UAE**: urinary albumin excretion.

## Competing interests

The authors declare that they have no competing interests.

## Authors' contributions

All authors contributed in the same way.
